# A Sensor Data Fusion System Based on *k*-Nearest Neighbor Pattern Classification for Structural Health Monitoring Applications

**DOI:** 10.3390/s17020417

**Published:** 2017-02-21

**Authors:** Jaime Vitola, Francesc Pozo, Diego A. Tibaduiza, Maribel Anaya

**Affiliations:** 1 Control, Dynamics and Applications (CoDAlab), Departament de Matemàtiques, Escola d’Enginyeria de Barcelona Est (EEBE), Universitat Politècnica de Catalunya (UPC), Campus Diagonal-Besòs (CDB), Eduard Maristany, 6–12, Sant Adrià de Besòs, Barcelona 08930, Spain; jaimevitola@usantotomas.edu.co; 2 Departamento de Ingeniería Eléctrica y Electrónica, Universidad Nacional de Colombia, Cra 45 No. 26-85, Bogotá 111321, Colombia; dtibaduizab@unal.edu.co; 3 Faculty of Engineering, Fundación Universitaria Los Libertadores, Cra 16 No. 63A-68, Bogotá 111221, Colombia; maribel.anaya@libertadores.edu.co; 4 MEM (Materials-Electronics and Modelling Research Group), Faculty of Electronics Engineering, Universidad Santo Tomás, Cra 9 No. 51-11, Bogotá 110231, Colombia

**Keywords:** piezoelectric, sensors, active system, data fusion, machine learning, damage classification

## Abstract

Civil and military structures are susceptible and vulnerable to damage due to the environmental and operational conditions. Therefore, the implementation of technology to provide robust solutions in damage identification (by using signals acquired directly from the structure) is a requirement to reduce operational and maintenance costs. In this sense, the use of sensors permanently attached to the structures has demonstrated a great versatility and benefit since the inspection system can be automated. This automation is carried out with signal processing tasks with the aim of a pattern recognition analysis. This work presents the detailed description of a structural health monitoring (SHM) system based on the use of a piezoelectric (PZT) active system. The SHM system includes: (i) the use of a piezoelectric sensor network to excite the structure and collect the measured dynamic response, in several actuation phases; (ii) data organization; (iii) advanced signal processing techniques to define the feature vectors; and finally; (iv) the nearest neighbor algorithm as a machine learning approach to classify different kinds of damage. A description of the experimental setup, the experimental validation and a discussion of the results from two different structures are included and analyzed.

## 1. Introduction

The service life of structures is affected by several factors, such as the quality of the materials and components, environmental effects, operational conditions and the quality of the building, among others. For these reasons, it is essential to inspect the structure during its service life. The revision and maintenance operation may depend on the kind of structure. However, in an automated monitoring system, some common elements are of interest, damage detection, localization and classification being some of the most important. The damage identification reliability is associated with the use of a reliable sensor network since faults in the sensors can lead to false positives in the damage detection process. Sensor fault or damage is commonly based on sensor debonding, piezoelectric fractures or bad connections, produced at the very moment of the installation of the monitoring system or during its lifetime. To detect these kinds of failures, several approaches have been developed, among them data-driven algorithms to detect crystal cuts and debonding at different temperatures [1], crystal removals [2], the effects of cracks and debonding in the usability of the signals for structural damage detection [3].

When it is possible to ensure the proper performance of the sensors, damage identification tasks can be applied. On this topic, it is possible to find some strategies for damage detection, localization and classification, including robust detection [4], which considers the variations in the environmental conditions; or even the use of a robust regression technique to analyze data from an SHM system in order to distinguish between damage and environmental conditions [5], the development of a methodology to remove the environmental effects from the SHM data by using principal component analysis and Hilbert–Huang transformation [6] or the use of adaptive kernel spectral clustering that detects damage in its initial stage [7]. With respect to the use of machine learning approaches, several strategies have been explored. For instance, He and Wang [8] use *k*-NN algorithm (*k*-nearest neighbor rule) for the fault detection in semiconductor manufacturing processes. Similarly, numerical and experimental investigations to compare metrics to assess and compensate the degradation of the adhesive layer of surface-bonded piezoelectric (lead zirconate titanate, PZT) transducers for SHM are performed in [9]. Other techniques include support vector machines [10], naive Bayes classifiers, feed-forward neural networks, random forest and AdaBoost [11], among others. This paper is not focused on the analysis of the sensor faults or the effects of the environmental condition in the damage identification process on the structural damage classification by means of a data-driven algorithm, which considers the use of data from healthy piezoelectric sensors in a sensor network permanently attached to the structure that has to be inspected.

Previous works by the authors include the use and development of multivariate analysis techniques, such as linear principal component analysis (PCA), non-linear PCA [12] and independent component analysis (ICA), to detect [13], classify and localize damage in structures [2]. In this paper, a smart system with data acquisition and data management is described. The system considers the use of a piezoelectric sensor network, multivariate analysis and machine learning. The proposed system presents new contributions since it introduces the use of a novelty sensor data fusion for data organization, the use of featured vectors and *k*-nearest neighbors machines, which allows one to detect and classify different kinds of damage.

The structure of the paper is as follows: In Section 2, a brief description of the theoretical background required to construct the SHM system is presented. Section 3 describes the SHM system that is used to inspect the structures and the strategies that are applied to classify the damage. In Section 4, the experimental setup is introduced together with some results. Finally, in Section 5, some concluding remarks are discussed.

## 2. Theoretical Background

### 2.1. Piezoelectric Sensors

Knowledge about changes in a system due to environmental or operational conditions is a requirement in modern control and monitoring systems. In this sense, it is necessary to be in possession of devices that can convert analog information—temperature, pressure, sound, acceleration, acoustic emission, among others—in electric information to be used in control or acquisition systems. Different kinds of sensors based on physical effects can be currently found. One of these is the piezoelectric sensors, which are transducers able to sense pressure, acceleration, temperature, strain of force and acoustic emission by means of the piezoelectric effect and convert this information into an electrical charge [14].

Some advantages of the inspection with piezoelectric transducers include high sensitivity to the damage, easy installation and operation, since relatively long distance inspection can be covered with low attenuation and reduced price, compared with other sensors. Additionally, these kinds of sensors can be used as passive or active sensors since they can work both as a sensor or as actuators. Some limitations in the use of piezoelectric transducers for inspection processes are, for instance, a low output. This means that it is required to use an additional circuit to amplify the excitation/collected signals and high impedance output [15].

### 2.2. Principal Component Analysis

One of the greatest difficulties in data analysis arises when the amount of data is very large and there is no apparent relationship between all of the information or when this relationship is very difficult to find. In this sense, principal component analysis was born as a very useful tool to reduce and analyze a big quantity of information. Principal component analysis was described for the first time by Pearson in 1901, as a tool of multivariate analysis and was also used by Hotelling in 1933 [16]. This method allows one to find the principal components, which are a reduced version of the original dataset and include relevant information that identifies the reason for the variation between the measured variables. To find these variables, the analysis includes the transformation of the data with respect to a current coordinate space to a new space in order to re-express the original data trying to reduce, filter or eliminate the noise and possible redundancies. These redundancies are measured by means of the correlation between the variables [17].

There are two mechanisms to implement the analysis of principal components: (i) the first method is based on correlations; and (ii) a second strategy that is based on the covariance. It is necessary to highlight that PCA is not invariant to scale, so the data under study must be normalized. Many methods can be used to perform this normalization, as is shown in [17,18]. In many applications, PCA is also used as a tool to reduce the dimensionality of the data. Currently, there are several useful toolbox that implement PCA and analyze the reduced data provided by this strategy [19]. For the sake of completeness, we present in the following sections a succinct description of the PCA modeling that includes how the measured data are arranged in matrix form. We also present the normalization procedure (group scaling) and how the new data to inspect are projected onto the PCA model.

#### 2.2.1. PCA Modeling

The first step to build a PCA model is to measure, from a healthy structure, different sensors or variables during (L−1)Δ seconds, where *Δ* is the sampling time, and n∈N experimental trials. The discretized measures of the sensors can be arranged in matrix form as follows:

(1) $$\begin{array}{cc} X & =\left(\begin{array}{cccc} x_{11}^{1} & x_{12}^{1} & \cdots & x_{1L}^{1}\\ \vdots & \vdots & \ddots & \vdots \\ x_{i1}^{1} & x_{i2}^{1} & \cdots & x_{iL}^{1}\\ \vdots & \vdots & \ddots & \vdots \\ x_{n1}^{1} & x_{n2}^{1} & \cdots & x_{nL}^{1} \end{array}|\begin{array}{ccc} x_{11}^{2} & \cdots & x_{1L}^{2}\\ \vdots & \ddots & \vdots \\ x_{i1}^{2} & \cdots & x_{iL}^{2}\\ \vdots & \ddots & \vdots \\ x_{n1}^{2} & \cdots & x_{nL}^{2} \end{array}|\begin{array}{c} \cdots \\ \ddots \\ \cdots \\ \ddots \\ \cdots \end{array}|\begin{array}{ccc} x_{11}^{N} & \cdots & x_{1L}^{N}\\ \vdots & \ddots & \vdots \\ x_{i1}^{N} & \cdots & x_{iL}^{N}\\ \vdots & \ddots & \vdots \\ x_{n1}^{N} & \cdots & x_{nL}^{N} \end{array}\right)\in M_{n\times (N\cdot L)}\left(R\right)\\ & =\left(\begin{array}{ccccccc} X^{1} & | & X^{2} & | & \cdots & | & X^{N} \end{array}\right) \end{array}$$

where $M_{n\times (N\cdot L)}\left(R\right)$ is the vector space of n×(N·L) matrices over R and N∈N is the number of sensors. It is worth noting that each row vector $X\left(i,:\right)\in R^{N\cdot L},\;i=1,\ldots ,n$ of matrix X in Equation (1) represents the measurements from all of the sensors at a given experimental trial. Similarly, each column vector $X\left(:,j\right)\in R^{n},\;j=1,\ldots ,N\cdot L,$ contains measurements from one sensor at one specific time instant in the whole set of experimental trials.

As stated before, one of the goals of PCA is to eliminate the redundancies in the original data. This objective is achieved through a linear transformation orthogonal matrix:

$$\begin{array}{c} P\in M_{\left(N\cdot L)\times (N\cdot L\right)}\left(R\right) \end{array}$$

that is used to transform or project the original data matrix X in Equation (1) according to the matrix product:

$$\begin{array}{c} T=\mathrm{XP}\in M_{n\times (N\cdot L)}\left(R\right) \end{array}$$

where the resulting matrix T has a diagonal covariance matrix.

#### 2.2.2. Normalization: Group Scaling

Since the data in matrix X come from several sensors and could have different magnitudes and PCA is not invariant to scale, a preprocessing stage must be applied to rescale the data. This normalization is based on the mean of all measurements of the sensor at the same time instant and the standard deviation of all measurements of the sensor. In this sense, for k=1,…,N, we define:

(2) $$\begin{array}{cc} \mu_{j}^{k} & =\frac{1}{n}\sum_{i=1}^{n}x_{ij}^{k},\;j=1,\ldots ,L, \end{array}$$

(3) $$\begin{array}{cc} \mu^{k} & =\frac{1}{nL}\sum_{i=1}^{n}\sum_{j=1}^{L}x_{ij}^{k}, \end{array}$$

(4) $$\begin{array}{cc} \sigma^{k} & =\sqrt{\frac{1}{nL}\sum_{i=1}^{n}\sum_{j=1}^{L}{\left(x_{ij}^{k}-\mu^{k}\right)}^{2},} \end{array}$$

where $\mu_{j}^{k}$ is the mean of the measures placed at the same column, that is the mean of the *n* measures of sensor *k* in matrix $X^{k}$ at time instants $\left(j-1\right)\Delta$ seconds; $\mu^{k}$ is the mean of all of the elements in matrix $X^{k}$, that is the mean of all of the measures of sensor *k*; and $\sigma^{k}$ is the standard deviation of all of the measures of sensor *k*. Then, the elements $x_{ij}^{k}$ of matrix X are scaled to define a new matrix $\overset{ˇ}{X}$ as:

(5) $$\begin{array}{cc} {\overset{ˇ}{x}}_{ij}^{k} & :=\frac{x_{ij}^{k}-\mu_{j}^{k}}{\sigma^{k}},\;i=1,\ldots ,n,\;j=1,\ldots ,L,\;k=1,\ldots ,N. \end{array}$$

For the sake of simplicity, the scaled matrix $\overset{ˇ}{X}$ is renamed again as X. One of the properties of the scaled matrix X is that it is mean-centered [20]. Consequently, the covariance matrix of X can be defined and computed as:

(6) $$\begin{array}{c} C_{X}=\frac{1}{n-1}X^{T}X\in M_{\left(N\cdot L)\times (N\cdot L\right)}\left(R\right). \end{array}$$

The subspaces in PCA are defined by the eigenvectors and eigenvalues of the covariance matrix as follows:

(7) $$C_{X}P=P\Lambda$$

where the columns of $P\in M_{\left(N\cdot L)\times (N\cdot L\right)}\left(R\right)$ are the eigenvectors of $C_{X}$ and are defined as the principal components. The diagonal terms of matrix $\Lambda \in M_{\left(N\cdot L)\times (N\cdot L\right)}\left(R\right)$ are the eigenvalues $\lambda_{i},\;i=1,\ldots ,N\cdot L,$ of $C_{X}$, whereas the off-diagonal terms are zero, that is,

(8) $$\begin{array}{cc} \Lambda_{ii} & =\lambda_{i},\;i=1,\ldots ,N\cdot L \end{array}$$

(9) $$\begin{array}{cc} \Lambda_{ij} & =0,\;i,j=1,\ldots ,N\cdot L,\;i\neq j \end{array}$$

The goal of principal component analysis is two-fold; on the one hand, to eliminate the redundancies of the original data. This is achieved by transforming the original data through the projection defined by matrix P in Equation (7). On the other, a second goal is to reduce the dimensionality of the dataset X. This second objective is achieved by selecting only a limited number ℓ<N·L of principal components related to the *ℓ* highest eigenvalues. In this manner, given the reduced matrix:

(10) $$\begin{array}{c} \hat{P}=\left(p_{1}|p_{2}\left|\cdots \right|p_{\ell }\right)\in M_{N\cdot L\times \ell }\left(R\right), \end{array}$$

matrix $\hat{T}$ is defined as:

(11) $$\begin{array}{c} \hat{T}=X\hat{P}\in M_{n\times \ell }\left(R\right). \end{array}$$

#### 2.2.3. Projection of New Data onto the PCA Model

The current structure to inspect is excited by the same signal as the one that excited the healthy one in Section 2.2.1. Therefore, when the measures are obtained from N∈N sensors during (L−1)Δ seconds and ν∈N experimental trials, a new data matrix Y is constructed as in Equation (1):

(12) $$\begin{array}{cc} Y & =\left(\begin{array}{cccc} y_{11}^{1} & y_{12}^{1} & \cdots & y_{1L}^{1}\\ \vdots & \vdots & \ddots & \vdots \\ y_{i1}^{1} & y_{i2}^{1} & \cdots & y_{iL}^{1}\\ \vdots & \vdots & \ddots & \vdots \\ y_{\nu 1}^{1} & y_{\nu 2}^{1} & \cdots & y_{\nu L}^{1} \end{array}|\begin{array}{ccc} y_{11}^{2} & \cdots & y_{1L}^{2}\\ \vdots & \ddots & \vdots \\ y_{i1}^{2} & \cdots & y_{iL}^{2}\\ \vdots & \ddots & \vdots \\ y_{\nu 1}^{2} & \cdots & y_{\nu L}^{2} \end{array}|\begin{array}{c} \cdots \\ \ddots \\ \cdots \\ \ddots \\ \cdots \end{array}|\begin{array}{ccc} y_{11}^{N} & \cdots & y_{1L}^{N}\\ \vdots & \ddots & \vdots \\ y_{i1}^{N} & \cdots & y_{iL}^{N}\\ \vdots & \ddots & \vdots \\ y_{\nu 1}^{N} & \cdots & y_{\nu L}^{N} \end{array}\right)\in M_{\nu \times (N\cdot L)}\left(R\right) \end{array}$$

It is worth noting, at this point, that the natural number *ν* (the number of rows of matrix Y) is not necessarily equal to *n* (the number of rows of X), but the number of columns of Y must agree with that of X; that is, in both cases, the number *N* of sensors and the number of time instants *L* must be equal.

Before the collected data arranged in matrix Y are projected into the new space spanned by the eigenvectors in matrix P in Equation (7), the matrix has to be scaled to define a new matrix $\overset{ˇ}{Y}$ as in Equation (5):

(13) $$\begin{array}{cc} {\overset{ˇ}{y}}_{ij}^{k} & :=\frac{y_{ij}^{k}-\mu_{j}^{k}}{\sigma^{k}},\;i=1,\ldots ,\nu ,\;j=1,\ldots ,L,\;k=1,\ldots ,N, \end{array}$$

where $\mu_{j}^{k}$ and $\sigma^{k}$ are the real numbers defined and computed in Equations (2) and (4), respectively.

The projection of each row vector $r^{i}=\overset{ˇ}{Y}\left(i,:\right)\in R^{N\cdot L},i=1,\ldots ,\nu$ of matrix $\overset{ˇ}{Y}$ into the space spanned by the eigenvectors in $\hat{P}$ is performed through the following vector to matrix multiplication:

(14) $$\begin{array}{c} t^{i}=r^{i}\cdot \hat{P}\in R^{\ell }. \end{array}$$

For each row vector $r^{i},i=1,\ldots ,\nu$, the first component of vector $t^{i}$ is called the first score or Score 1; similarly, the second component of vector $t^{i}$ is called the second score or Score 2, and so on.

### 2.3. Machine Learning

Machine learning has revolutionized the way that complex problems have been tackled with the help of computer programs. In the incessant and relentless pursuit of the best tools for data analysis, machine learning has been highlighted for its capability for providing a quite remarkable set of strategies for pattern recognition. More precisely, when a deterministic mathematical model is difficult to define and data have, at first glance, no correlation, these pattern recognition techniques are generally able to find some kind of relationship. Machine learning strategies and bio-inspired algorithms allow one to avoid this difficulty through mechanisms designed to find the answer by themselves. In SHM or related areas, it is possible to find some applications about how machine learning has been used to detect problems, such as breaks, corrosion, cracks, impact damage, delamination, disunity and breaking fibers (some pertinent to metals and the others to composite materials) [21]. In addition, machine learning has been also used to provide information about the future behavior of a structure under extreme events, such as earthquakes [22].

**Figure 1:**
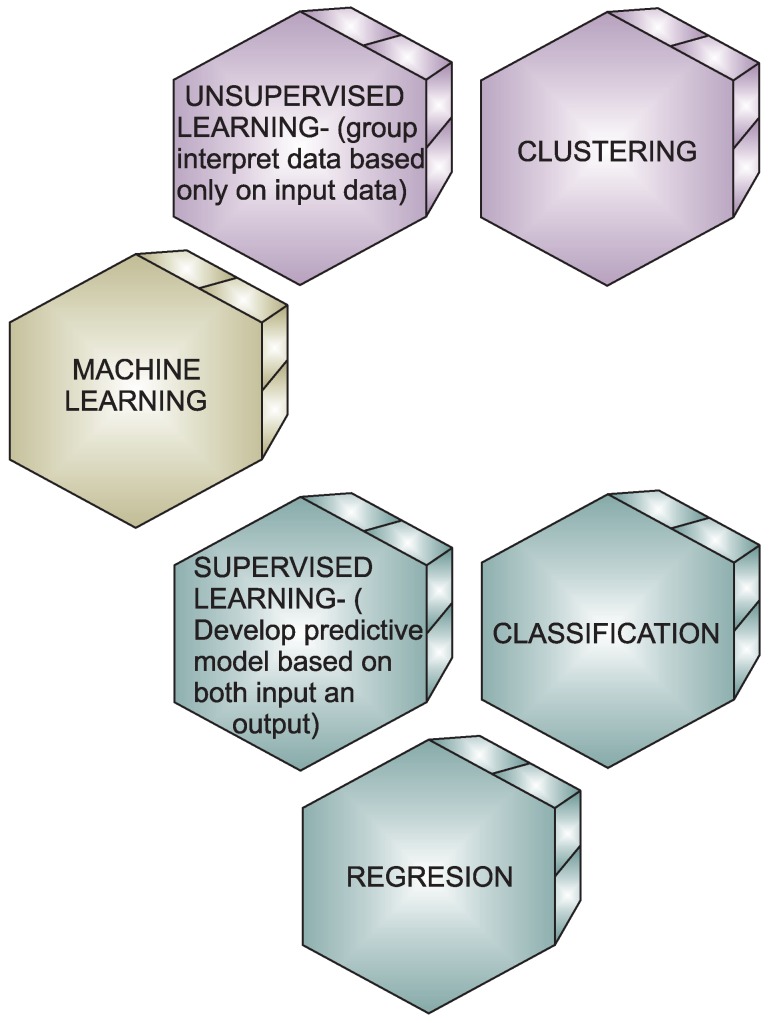
Classification of the machine learning approaches according to the learning.

Depending on how the algorithms are implemented, machine learning can be classified in two main approaches: unsupervised and supervised learning. In the first case, the information is grouped and interpreted using the input data uniquely. However, to perform the learning task in the second case, information about the output data is required. Figure 1 shows this classification and includes information about the kind of tasks that can be performed—clustering, classification, regression.

This paper is focused on the use of a supervised learning approach and, particularly, on the use of nearest neighbor classification. A brief description of the nearest neighbor pattern classification is introduced in the following subsection.

### 2.4. Nearest Neighbor Pattern Classification

The nearest neighbor (NN) is a simple nonparametric and highly efficient technique [23] that has been used in several areas, such as pattern recognition, ranking models or text categorization and classification for big data [24,25], just to name a few. One of the most used algorithms in machine learning applications is the *k*-NN, also known as *k*-nearest neighbors. *k*-NN is outstanding due to its simplicity, and the excellent results obtained when this technique is applied to diverse problems [26]. This algorithm works by using an input vector with the *k* closest training samples in the feature space. To perform the classification, the algorithm identifies the most common class among the *k* nearest neighbors. The algorithm requires a training to define the neighbors based on the distance from the test sample and a testing step to determine the class to which this test sample belongs [26].

The number of neighbors can be changed to adjust the *k*-NN algorithm. In this sense, for instance, the use of one neighbor is known as fine *k*-NN, and a coarse *k*-NN uses 100 neighbors. Many neighbors can be time consuming to fit. There are six different *k*-NN classifiers available in MATLAB that can be used to classify data [27], and these classifiers are based on different distances. Some of them—fine, medium and coarse *k*-NN algorithms—make use of the Euclidean distance to determine the nearest neighbors. According to MATLAB, each classifier works as follows [26]:

- Fine *k*-NN: A nearest neighbor classifier that makes finely detailed distinctions between classes with the number of neighbors set to one.
- Medium *k*-NN: A nearest neighbor classifier with fewer distinctions than a fine *k*-NN with the number of neighbors set to 10.
- Coarse *k*-NN: A nearest neighbor between classes, with the number of neighbors set to 100.
- Cosine *k*-NN: A nearest neighbor classifier that uses the cosine distance metric. The cosine distance between two vectors *u* and *v* is defined as:
  $$\begin{array}{c} 1-\frac{u\cdot v}{\left|u|\cdot |v\right|}, \end{array}$$
  that is, one minus the ratio of the inner product of *u* and *v* over the product of the norms of *u* and *v*.
- Cubic *k*-NN: A nearest neighbor classifier that uses the cubic distance metric. The cubic distance between two *n*-dimensional vectors *u* and *v* is defined as:
  $$\begin{array}{c} \sqrt[{3}]{\sum_{i=1}^{n}{\left|u_{i}-v_{i}\right|}^{3}}. \end{array}$$
- Weighted *k*-NN: A nearest neighbor classifier that uses distance weighting. The weighted Euclidean distance between two *n*-dimensional vectors *u* and *v* is defined as:
  $$\begin{array}{c} \sqrt{\sum_{i=1}^{n}w_{i}{\left(x_{i}-y_{i}\right)}^{2}}, \end{array}$$
  where $0<w_{i}<1$ and $\sum_{i=1}^{n}w_{i}=1$.

*k*-NN has been used successfully in fault detection for gas sensor arrays [25], classification for big data [28], fault detection and classification for high voltage DC transmission lines [26] and traffic state prediction [29], among others.

## 3. Structural Health Monitoring System

### 3.1. Hardware of the SHM System

**Figure 2:**
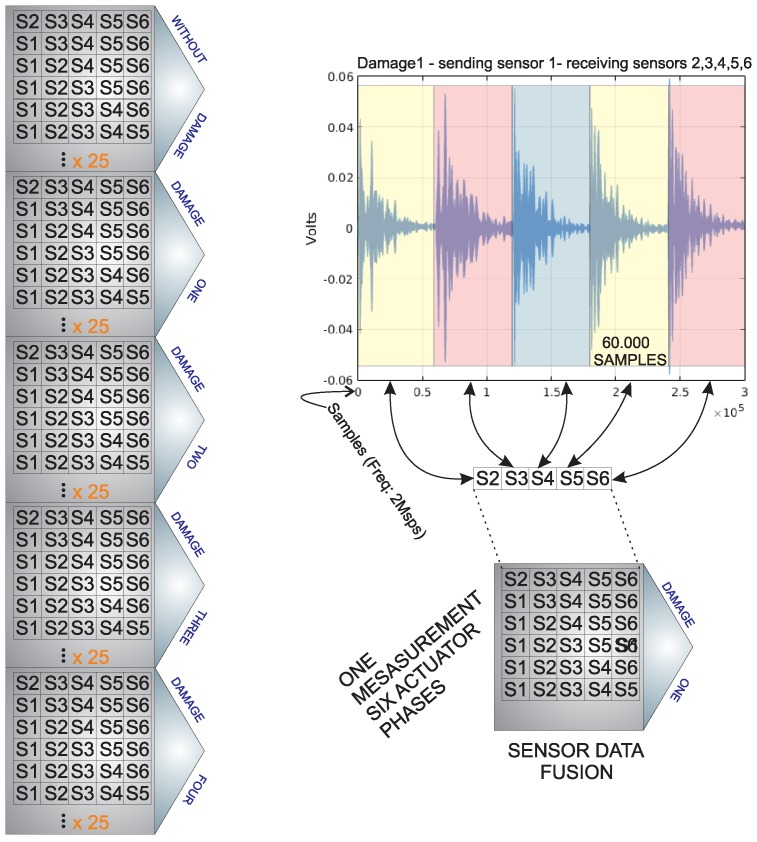
Data organization for sensor data fusion.

**Figure 3:**
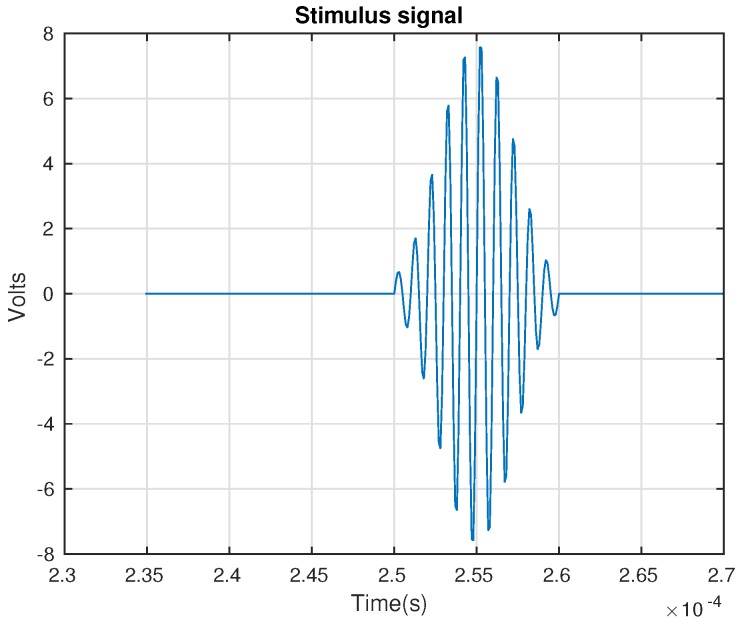
Excitation signal applied to the piezoelectric acting as the actuator, in each actuation phase.

The inspection system considers the use of a sensor network that is distributed on the surface of the structure. In this work, piezoelectric sensors are used. However, the methodology that is is introduced here is suitable for several kinds of vibration sensors. This is because the system considers the use of a baseline with signals from the structure in a healthy state, and the analysis is performed by the comparison of the new experiments under the same conditions (guided waves) with the baseline. The piezoelectric sensor network works in several actuation phases. Each actuation phase is defined by the use of a PZT as the actuator, and the rest of the piezoelectrics are used as sensors. This information is collected and organized in a matrix per actuator. Therefore, the measured signals are organized from Sensor 1 to sensor *N* for *N* sensors as can be seen in Figure 2. To this goal, a Tie Pie (HS5) is used and a signal, as in Figure 3, is applied. This signal is defined because it has a collection of signals in a reduced bandwidth that ensures that the acquired signal does not have as many components so as to make it difficult to hide the damage. The specifications of the signal are: 8 V of amplitude and a frequency of 10 kHz.

**Figure 4:**
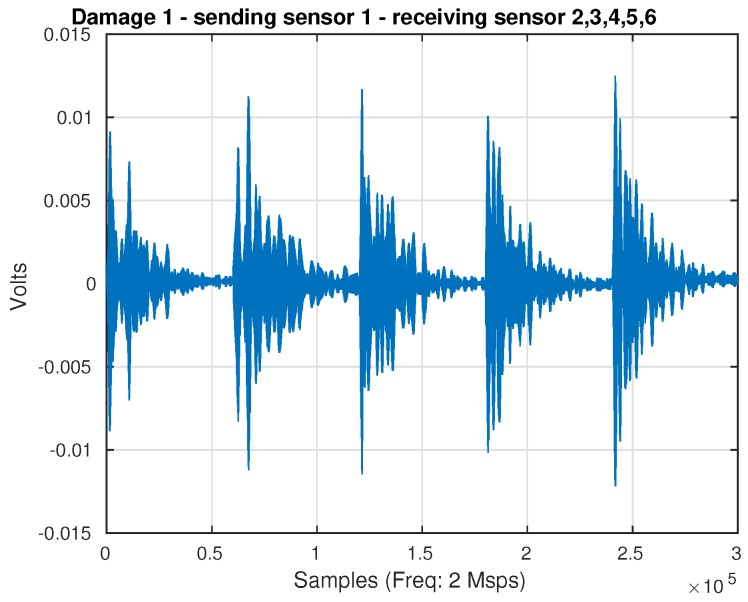
Received signals in the actuation Phase 1 when Damage 1 is performed on the structure.

Figure 4 presents the captured and organized signals for an aluminum plate instrumented with six sensors. It shows the actuation Phase 1 (PZT 1 as the actuator and the rest of the PZTs as sensors). These signals are captured by two oscilloscopes from Tie Pie company (HS4) from each sensor at a rate of up to two millions samples per second, and each channel contributes with signals of 60,000 samples. Figure 4 shows the result of the organization in the pre-processing step. As can be observed, there is a continuous signal that corresponds to the concatenation of the five signals measured by the PZT acting as sensors.

**Figure 5:**
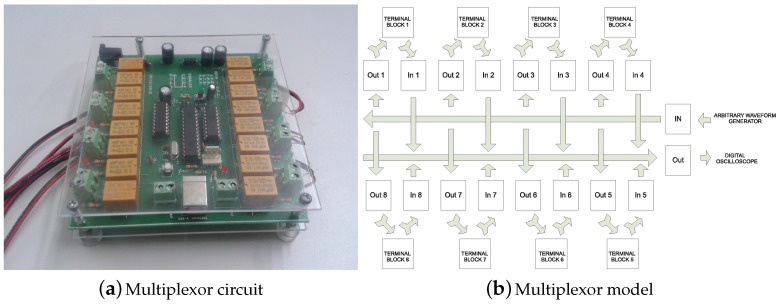
Multiplexor system used in the data acquisition system.

Due to the fact that the system only considers the use of an arbitrary waveform generator (HS5) with one channel, a multiplexer card was developed (Figure 5). This system works by connecting the analog input with one of the analog outputs defined by software.

Similarly, with the multiplexer card, it is possible to provide a direct way to the digitizers, which are, also in this case, from the company Tie Pie and with reference HS04. These devices are four-channel oscilloscopes with 14-bit resolution. In this work, two devices are used to involve eight channels. However and depending on the necessities, it is possible to add more of these devices.

**Figure 6:**
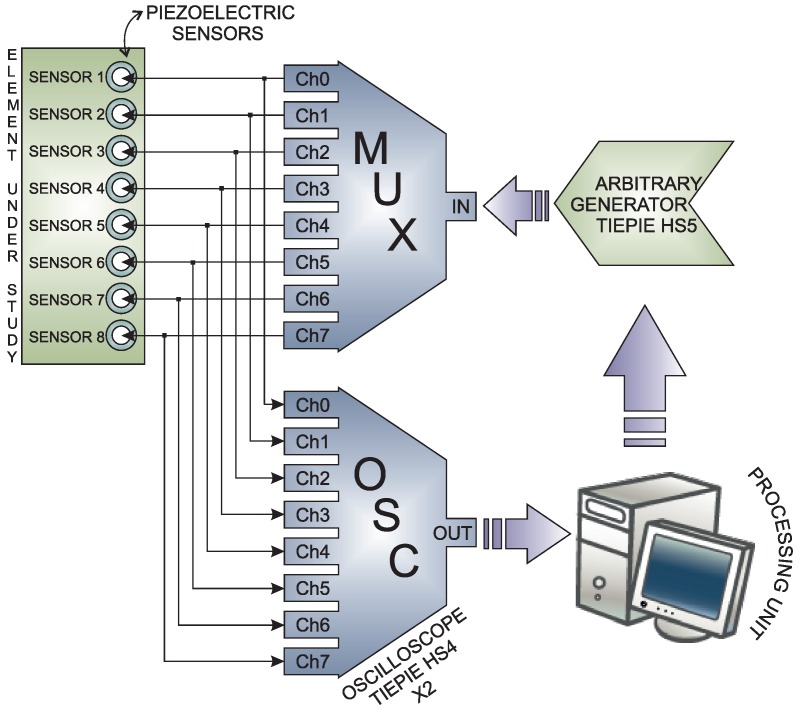
Representation of the structural health monitoring (SHM) system.

**Figure 7:**
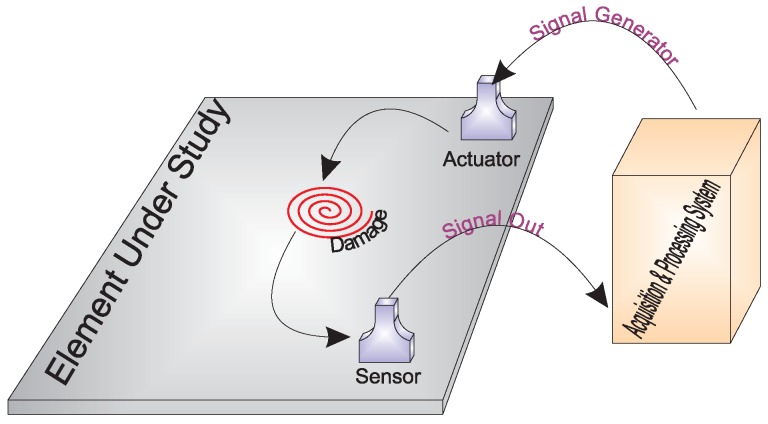
General scheme of the SHM system.

Figure 6 shows the general schema of the hardware in the SHM system. To sum up, the system defines one PZT as the actuator; the arbitrary wave generator applies a known signal (Figure 7); then, the signal is converted into a mechanical wave (lamb waves) and transferred to the structure. This wave travels superficially all across the structure interacting with the damage and the elements presented on the surface. The sensors convert the mechanical wave into an electric signal, and the digitizer collects the signals propagated though the structure in the rest of the sensors. Depending on the kind of structure, the system may require a power amplifier to amplify the signals applied to the actuators and to ensure good captured information.

### 3.2. Software of the SHM System

**Figure 8:**
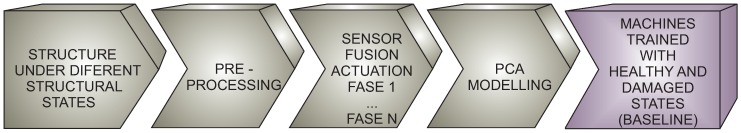
Training process with the data from the structure under different structural states.

The methodology is based on a pattern recognition perspective. In this sense, the strategy is considered to have two different steps. On the one hand, in the first step, a pattern is developed with the signals from the structure in healthy and damaged states, as is shown in Figure 8. To do that, the collected signals are pre-processed and organized by each actuation phase as was previously explained. These signals require pre-processing in order to be comparable because the data come from different places of the structure and are acquired with different amplitude values. In this case, group-scaling normalization is applied as detailed in Section 2.2.2. To define the pattern or the baseline, a feature vector is obtained by each actuation phase. A huge number of possible features can be extracted from the signals. In particular, the use of multivariate methods such as principal component analysis has proven to be very useful to perform this task. In the classification process and since *k*-NN is a supervised learning algorithm, different known damage and data from the healthy state are used to train the *k*-NN machines.

**Figure 9:**
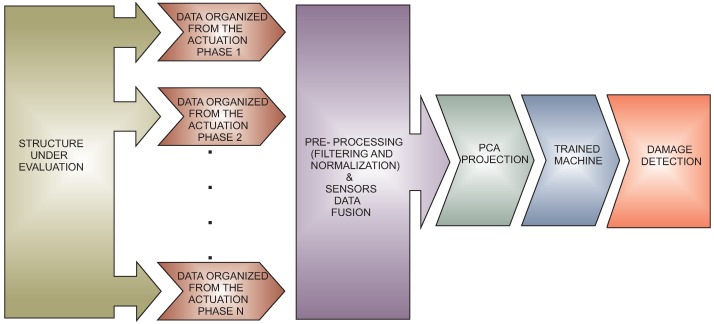
Testing step with data from the structure in an unknown structural state.

On the other hand, the second step corresponds to the testing. In this phase, the trained maps are used as a pattern, whereas experiments from the structure in unknown states are used to classify the current state of the structure, as is shown in Figure 9. The procedure to acquire and process the information is the same as in the development of the pattern. That is, the system digitizes the information from the sensors, and thereafter, the data are organized and pre-processed. In order to reduce the noise and to normalize the data, a Savitzky–Golay filter is applied. Subsequently the sensor data fusion is applied to organize the information by each actuation phase. Finally, principal component analysis is applied, and the resulting projections are used to define the feature vectors that will be the inputs to the machine learning approach.

**Figure 10:**
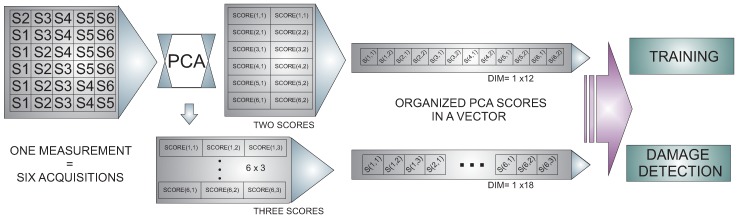
Feature vector organization.

The sensor data fusion takes place also in two steps: In the first stage, data acquisition and organization, a single PZT is used as an actuator, and the data collected from the rest of the piezoelectric transducers installed in the structure are used and organized in a vector. With this strategy, the information on how the sensors sense the damage, by each actuation phase, is available. In the second stage, an experiment from each actuation phase is extracted and organized in a matrix, as shown in Figure 10. After that, PCA is applied to this matrix to obtain a reduced version of these data, which is organized in a vector and submitted to a machine learning algorithm. Assembling the feature vectors by each actuation phase and the use of these vectors in the machine allows one to analyze the information from all of the actuation phases in a single machine. This process allows a reduction in the number of variables or figures that need to be analyzed or to organize all of the information.

**Figure 11:**
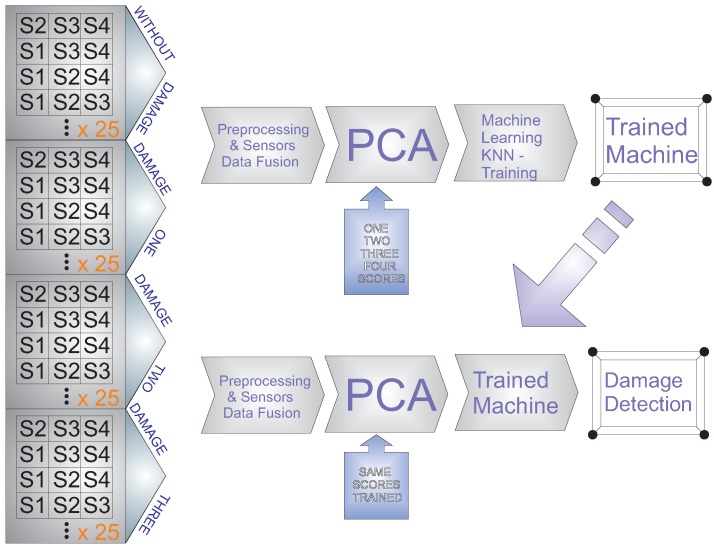
Data organization and testing step for damage detection and classification.

Figure 11 provides a general outline of the training process and the testing step (online execution or off-line execution). The system has the capability to detect and classify damage in off-line mode. To work in this mode, the state is stored in a file, an the software loads this information to apply the methodology. Otherwise, the system works in online mode when the data are acquired and analyzed, and the result of the evaluation is provided in a short time.

A distinguishing feature of the work that is presented in this paper with respect to previous works [18,30] is the way the data are organized and arranged. With the data organization as in Figure 2, we provide the structural health monitoring system all of the sensor data fusion that includes information from all of the actuation phases. More precisely and with respect to Figure 2, a structure instrumented with six piezoelectric transducers is considered. As can be observed in the left side of this figure, five structural states are considered: four different kinds of damage and the healthy state. Since there are six actuation phases, these phases are used to build five matrices corresponding to each structural state. Each matrix is organized as follows: the first row contains the information from the actuation Phase 1; the second row include the information from the actuation Phase 2; and so on for the rest of the actuation phases. In this case, 25 experiments were performed by each structural state by each actuation phase. Consequently, each matrix consists of a number of rows equal to 25 experiment × 6 actuation phases =150 rows and 5 columns. It is necessary to highlight that each column contains the collected samples from each sensor.

## 4. Experimental Setup and Results

**Figure 12:**
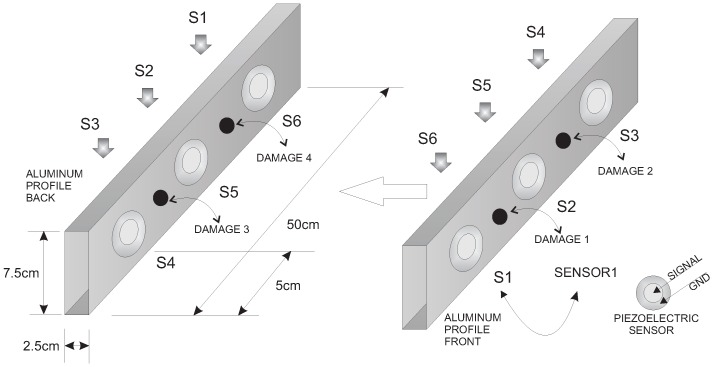
Aluminum rectangular profile instrumented with six piezoelectric sensors.

In this paper, three specimens (structures) are used to demonstrate the feasibility of the structural health monitoring system introduced in Section 3. These three specimens are:

(i) An aluminum rectangular profile with a sensor network formed by six piezoelectric transducers bonded on both sides of the profile; see Figure 12;
(ii) An aluminum plate with four piezoelectric transducers;
(iii) Composite plate of carbon fiber polymer with six piezoelectric transducers;

These structures have a different shape. Besides, both the size and number of piezoelectric transducers installed in theses structures and the number and location of the damage are different.

Damage was simulated by adding a mass to the structure in different positions, and 25 experiments were performed by each case (structural state), interpreting case as Damage 1, Damage 3, Damage 3, Damage 4 or the absence of damage.

The classification is performed considering six classifiers from the MATLAB Statistics and Machine Learning Toolbox:

- Fine *k*-NN
- Medium *k*-NN
- Coarse *k*-NN
- Cosine *k*-NN
- Cubic *k*-NN
- Weighted *k*-NN

We consider this selection of classifiers since these kind of machines are recommended to solve problems with data as those used in this paper. For this reason, this toolbox is used to train the machines, so the number of *k*-nearest neighbors is defined by the different classifiers as detailed in Section 2.4.

### 4.1. First Specimen: Aluminum Rectangular Profile

The first specimen that we consider in this paper is an aluminum rectangular profile that is instrumented with six piezoelectric sensors. The distribution of the piezoelectric transducers and the size and geometry of the specimen are shown in Figure 12. This figure also specifies the position of the four damage.

**Figure 13:**
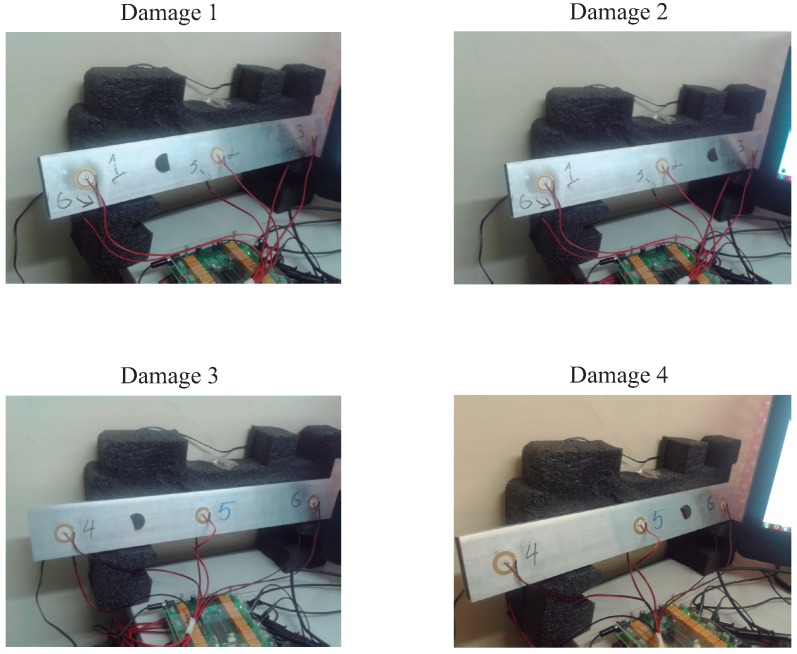
Aluminum rectangular profile instrumented with six piezoelectric sensors and with four different damage.

Figure 13 shows a composition of photography of the experiment where the four different kind of damage can be observed. As can also be seen from the pictures, the specimen is isolated from the noise and vibration, from different sources, that affect the laboratory. Isolation from possible external perturbations is critical since the noise and vibration could lead the structural health monitoring system to erroneous results.

**Figure 14:**
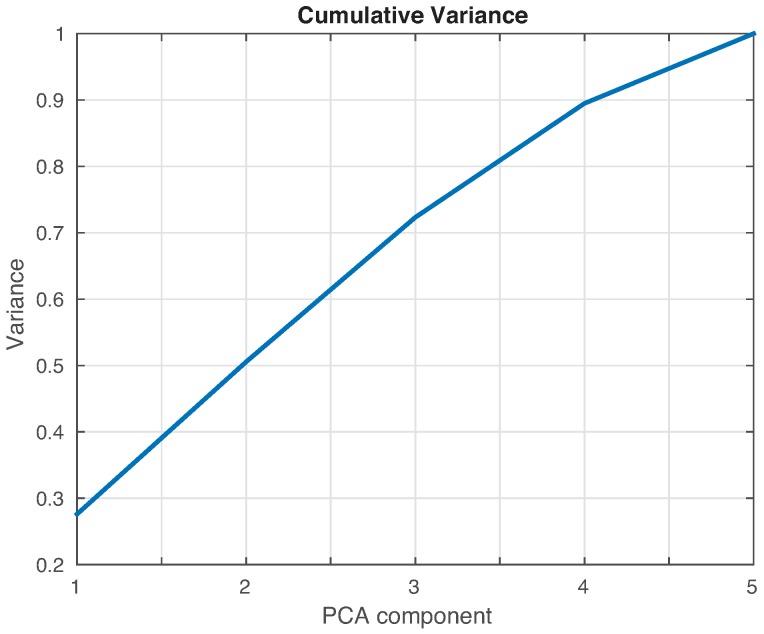
Cumulative contribution rate of variance accounted for the principal components.

The feature vector is formed by the projections or scores of the original data into the PCA model created as described in Section 2.2.1 and illustrated in Figure 10. The performance of the machines will be compared as a function of the number of scores that are considered. In general, the number of scores that have to be used depends on the cumulative contribution of variance that it is accounted for. More precisely, if the *i*-th score is related to the eigenvector $p_{i}$, defined in Equation (10), and the eigenvalue $\lambda_{i}$, in Equation (8), the cumulative contribution rate of variance accounting for the first σ∈N scores is defined as:

(15) $$\begin{array}{c} \frac{\sum_{i=1}^{\sigma }\lambda_{i}}{\sum_{i=1}^{\ell }\lambda_{i}}, \end{array}$$

where ℓ∈N is the number of principal components. In this sense, the cumulative contribution of the first five scores is depicted in Figure 14. It can be seen that the first two principal components account for 50% of the variance, while the first three principal components account for almost 75%, and the first four account for 90%. A priori, better results should be obtained if we use as many principal components as possible. However, in some cases, as reported in [31,32], less principal components may lead to more accurate results.

**Figure 15:**
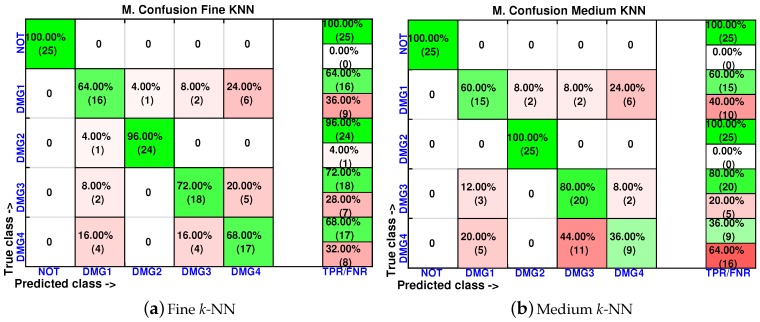
Confusion matrix using fine *k*-NN and medium *k*-NN when the feature vector is formed by the first principal component.

**Figure 16:**
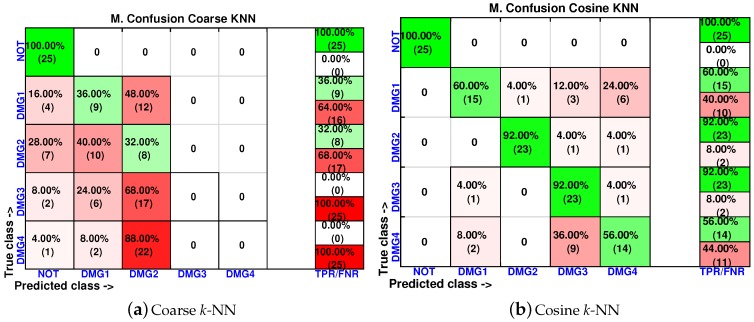
Confusion matrix using coarse *k*-NN and cosine *k*-NN when the feature vector is formed by the first principal component.

**Figure 17:**
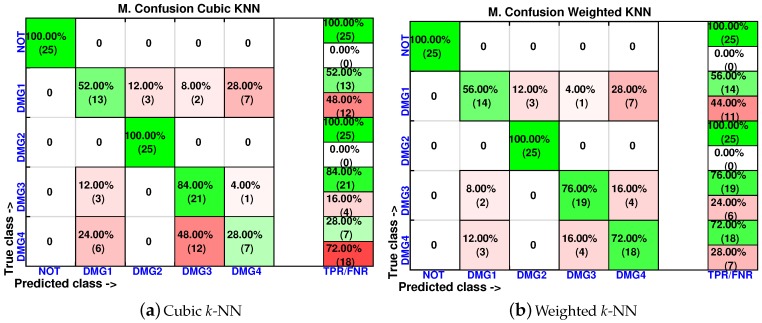
Confusion matrix using cubic *k*-NN and weighted *k*-NN when the feature vector is formed by the first principal component.

Figure 15, Figure 16 and Figure 17 show the classification results per machine or classifier where just the first score is used in the training process. These results include experiments where the damages are slightly displaced with respect to the original placement of the damage. The results with maximum accuracy in the classification are obtained when considering the weighted *k*-NN, the fine *k*-NN and the cosine *k*-NN classifiers. For instance, in the weighted *k*-NN classifier, 101 cases have been correctly classified out of 125 cases. This magnitude represents 81% of correct decisions. It is worth noting that, in all of the machines, the specimen with no damage is correctly classified in the totality of the cases. Similarly, all of the machines are able to separate the structure with no damage with respect to the structure with damage, with the exception of the coarse *k*-NN. In this case, the coarse *k*-NN fails to distinguish between the structure with damage and the structure with no damage in 14 out of 100 cases.

**Figure 18:**
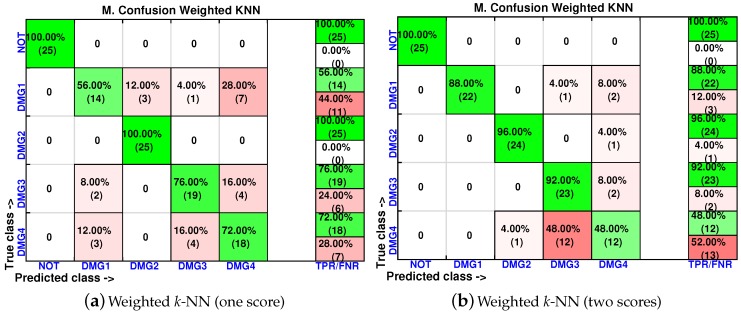
Confusion matrix using weighted *k*-NN when the feature vector is formed by the first principal component (**a**) or by the first two principal components (**b**).

**Figure 19:**
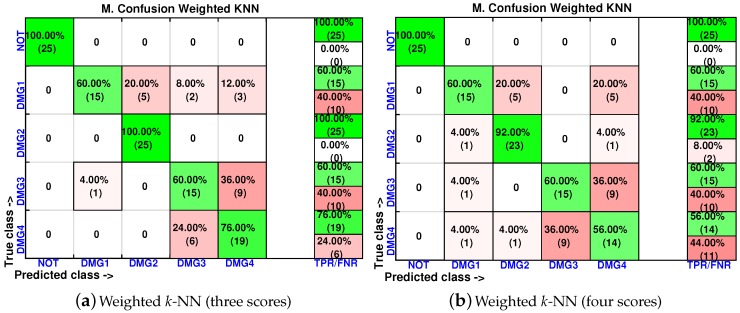
Confusion matrix using weighted *k*-NN when the feature vector is formed by the first three principal component (**a**) or by the first four principal components (**b**).

**Figure 20:**
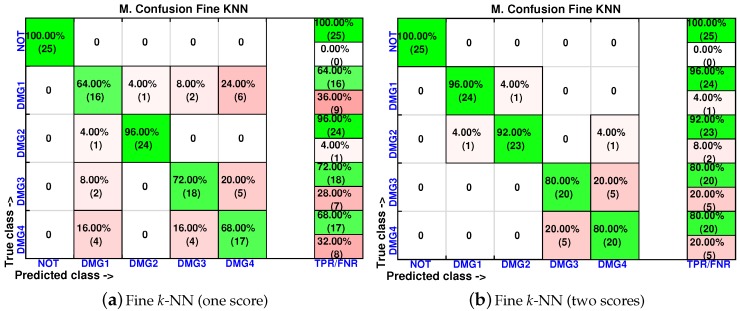
Confusion matrix using fine *k*-NN when the feature vector is formed by the first principal component (**a**) or by the first two principal components (**b**).

**Figure 21:**
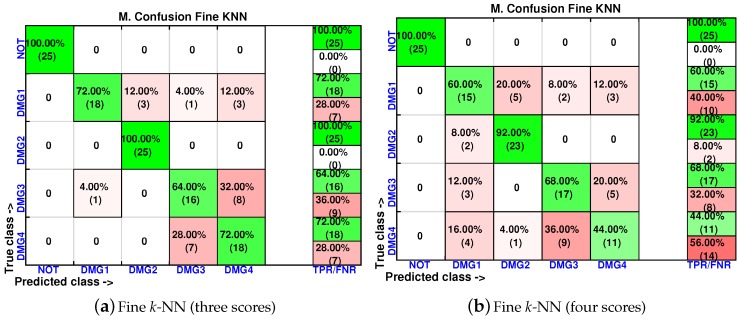
Confusion matrix using fine *k*-NN when the feature vector is formed by the first three principal component (**a**) or by the first four principal components (**b**).

In order to analyze the effect of the inclusion of more scores in the feature vector, the confusion matrices are calculated again for two of the best classifiers (weighted *k*-NN and fine *k*-NN) and with feature vectors of one, two, three and four scores. The results for the weighted *k*-NN classifier can be found in Figure 18 and Figure 19, whereas those corresponding to the fine *k*-NN classifier are summarized in Figure 20 and Figure 21.

It may seem surprising that the best results are obtained in this case when just two scores are used to define the feature vector. More precisely, in the weighted *k*-NN classifier, 106 cases have been correctly classified out of 125 cases, while in the fine *k*-NN classifier, this number rises up to 112 cases. This represents 85% and 90% of correct decisions, respectively. It is also worth noting that in the eight different scenarios that we have considered (two classifiers and four different sizes of the feature vector), the structure with no damage is correctly classified in the whole set of experiments. Similarly, the structure with damage is never confused with the structure with no damage. This means that the errors that appear in the classification are only due to a mistake in the identification of the damage.

**Figure 22:**
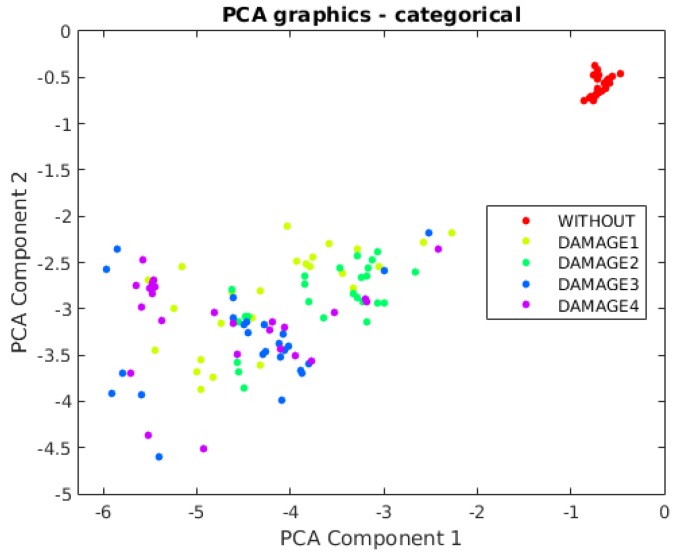
First principal component versus second principal component in the aluminum rectangular profile described in Section 4.1.

The first principal component versus the second principal component is depicted in Figure 22. It can be observed that a clear separation exists between the structure with no damage and the structure with the different kinds of damage. This is one of the reasons for the fact that the classifier performs quite well in terms of damage detection. However, from this figure, it is not possible to separate or classify the different damage, therefore showing the clear benefit of the use of a machine learning approach.

Finally, and back to the issue of the number of principal components that are used to define the feature vector, Mujica et al. [31] have already observed that, sometimes, the second principal component is often more effective to obtain accurate results in the damage detection or classification, contrary to what is expected. Similarly, an excessive number of principal components used to define the feature vector may lead to less good results since the SHM system may insert in the system part of the noise that we are trying to avoid.

### 4.2. Second Specimen: Aluminum Plate

**Figure 23:**
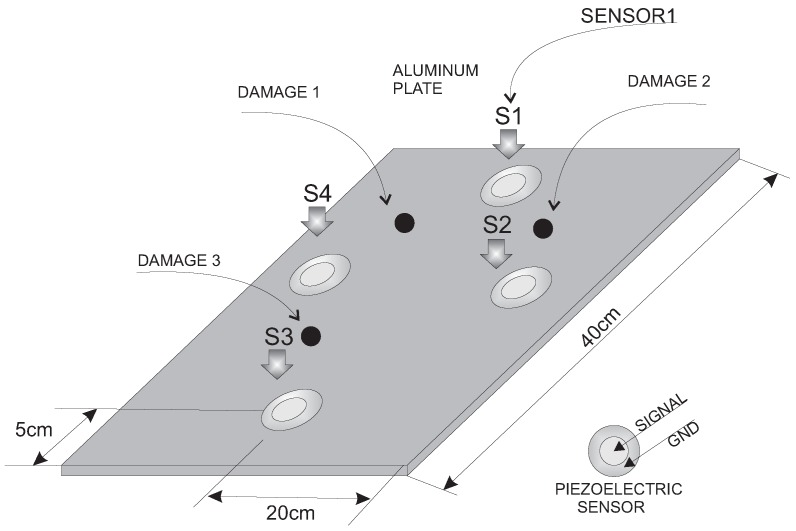
Aluminum plate instrumented with four piezoelectric sensors.

The second experimental validation was performed using an aluminum plate with an area of 40 cm × 40 cm and instrumented with four piezoelectric sensors, as shown in Figure 23. This figure also indicates the location of the three damages that are presented in the structure.

**Figure 24:**
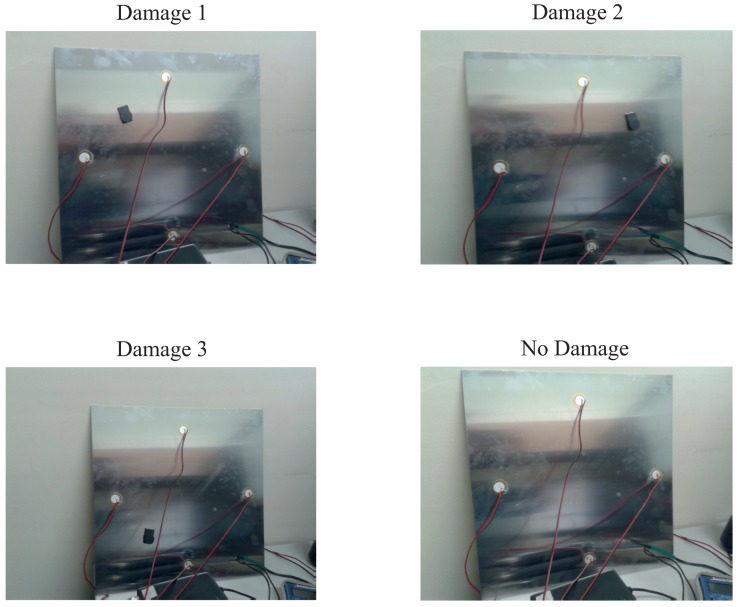
Experimental setup for the aluminum plate instrumented with four piezoelectric sensors and with different kind of damage.

Figure 24 shows a composition of the photography of the experiment where the three different kinds of damage and the structure with no damage can be observed. As can be seen from the pictures, the specimen is also isolated from the noise and vibration that affect the laboratory.

**Figure 25:**
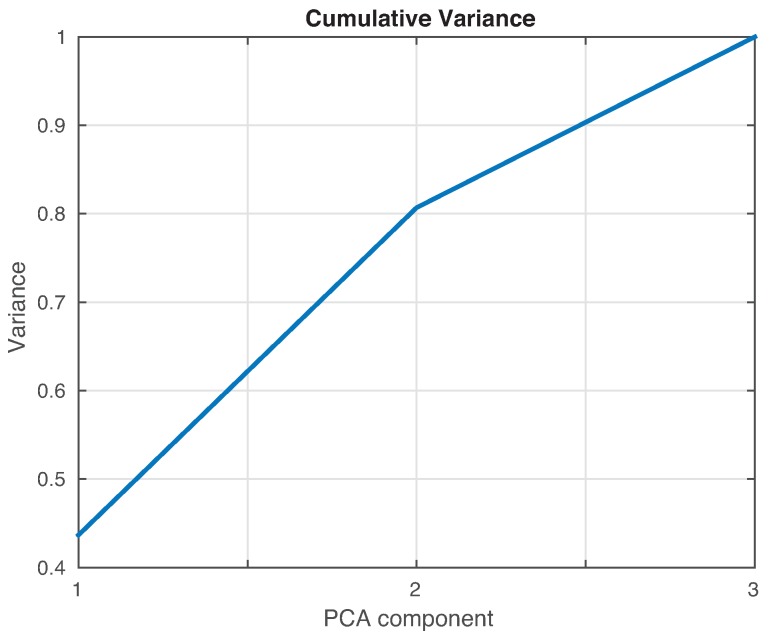
Cumulative contribution rate of variance accounting for the principal components from the data acquired from the aluminum plate.

**Figure 26:**
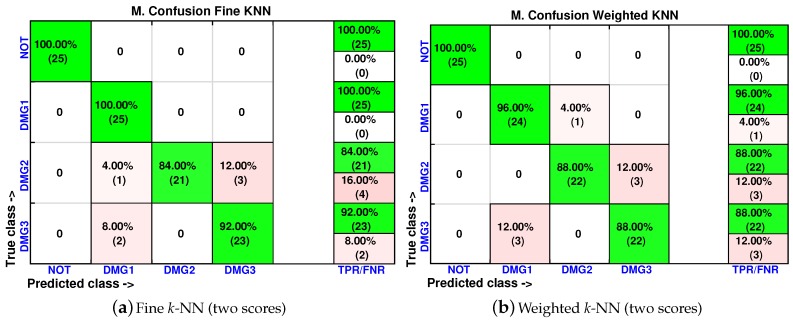
Confusion matrix using fine *k*-NN (**a**) and weighted *k*-NN (**b**) when the feature vector is formed by the first two principal components.

As in Section 4.1, the cumulative contribution of the first three scores is depicted in Figure 25. It can be seen that the first two principal components account for 82% of the variance, so we will use in this case the first two principal components’ analysis to create the feature vector. Figure 26 show the classification result for the fine *k*-NN and the weighted *k*-NN machines. In both classifiers, 93 cases have been correctly classified out of 100 cases. Besides, as with the previous specimen, the aluminum plate with no damage is correctly classified in the totality of the cases, and no confusion is made between the structure with no damage and the structure with damage.

**Figure 27:**
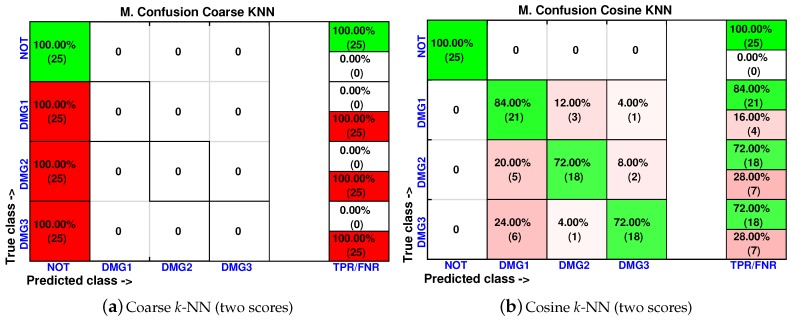
Confusion matrix using coarse *k*-NN (**a**) and cosine *k*-NN (**b**) when the feature vector is formed by the first two principal components.

We have also included, in Figure 27, the machines with the lowest accuracy in the classification. These classifiers are the coarse *k*-NN and the cosine *k*-NN. Although the percentage of correct decisions fluctuates between 72% and 100%, the cosine *k*-NN machine is still able to accurately identify the structure with no damage, and coarse *k*-NN had the worst performance.

**Figure 28:**
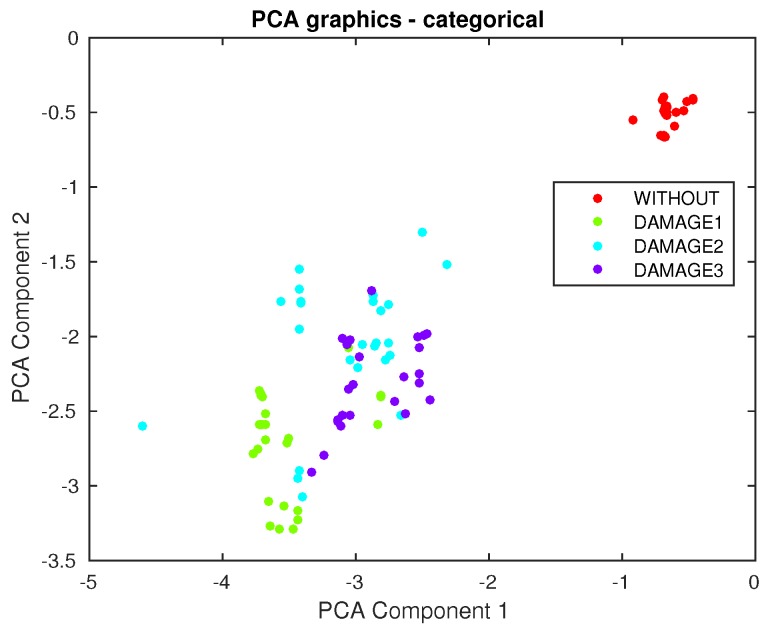
First principal component versus second principal component in the aluminum plate described in Section 4.2.

Furthermore, in this case, the first principal component versus the second principal component is depicted in Figure 28. It can be observed that a clear separation exists between the aluminum plate with no damage and the plate with the different kinds of damage. However, from this figure, it is not possible to separate or classify the different damage, therefore showing the clear benefit of the approach used in this work.

### 4.3. Third Specimen: Composite Plate, Carbon Fiber

**Figure 29:**
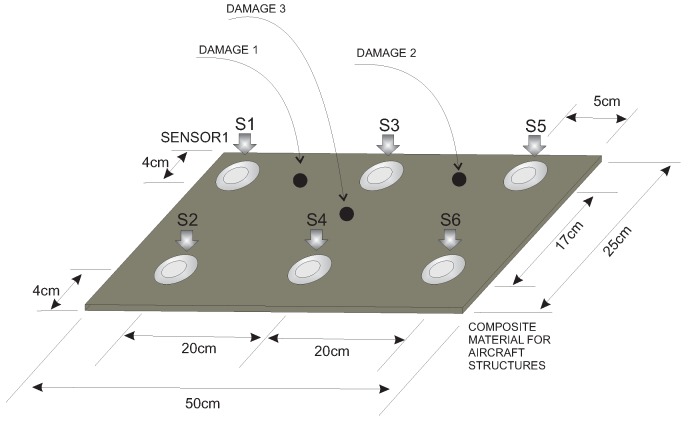
Composite plate instrumented with six piezoelectric sensors.

The third specimen used for the experimental validation of the approach presented in this paper is a composite plate of carbon fiber polymer with an area of 50 cm × 25 cm and a 2-mm thickness. The plate is instrumented with six piezoelectric transducers as shown in Figure 29. The figure also illustrates the location of the three damages that are placed in the structure.

**Figure 30:**
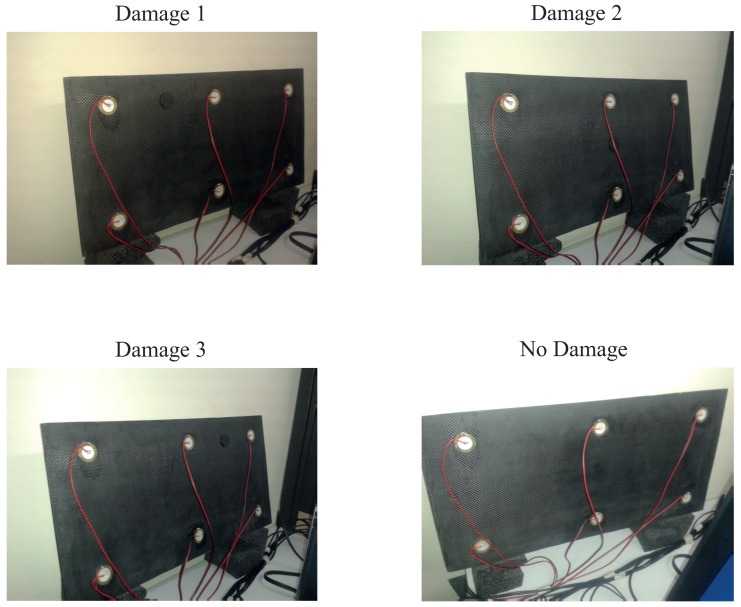
Experimental setup for the composite plate instrumented with six piezoelectric sensors.

Figure 30 shows a composition of images of the experiment with the distribution of the sensors and the vibration isolation similar to that of the previous specimens.

**Figure 31:**
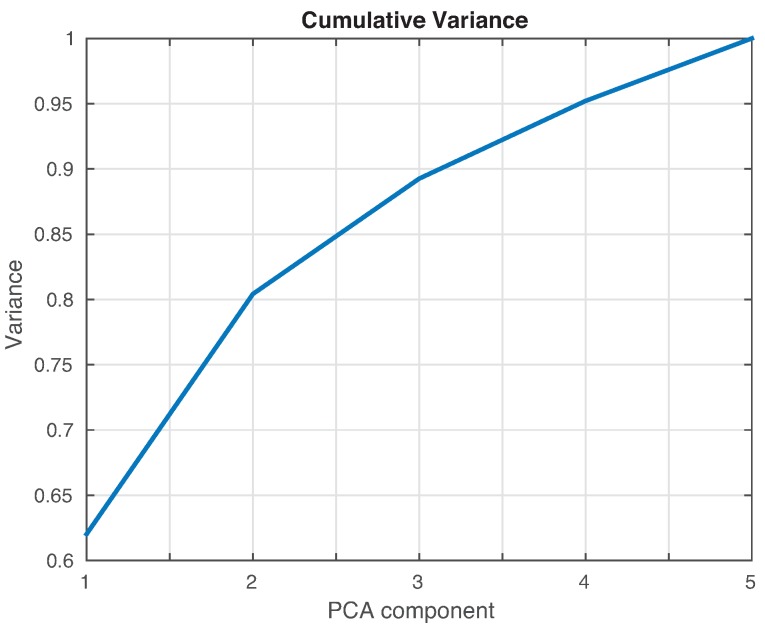
Cumulative contribution rate of variance accounted for the principal components from the data acquired from the composite plate.

**Figure 32:**
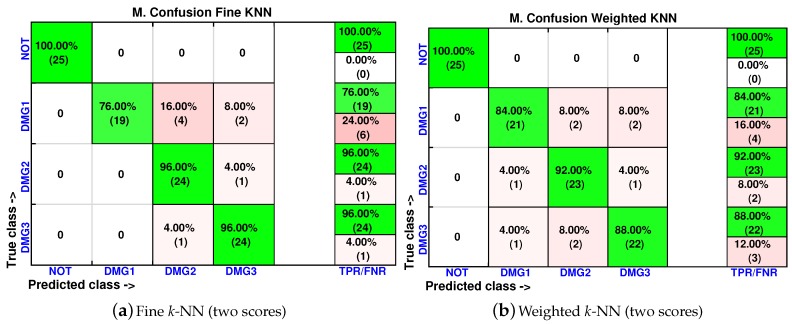
Confusion matrix using fine *k*-NN (**a**) and weighted *k*-NN (**b**) when the feature vector is formed by the first two principal components.

As in Section 4.1 and Section 4.2, the cumulative contribution of the first three scores is depicted in Figure 31. It can be seen that the first two principal components account for about 80% of the variance, so we will use again in this case the first two principal components’ analysis to create the feature vector. Figure 32 shows the classification result for the fine *k*-NN and the weighted *k*-NN machines. In both classifiers, 92 and 91 cases have been correctly classified out of 100 cases, respectively. These results are consistent with the results in Section 4.1 and Section 4.2 since these two classifiers present the best accuracy in the classification. Besides, as with the previous specimen, the aluminum plate with no damage is correctly classified in the totality of the cases, and no confusion is made between the structure with no damage and the structure with damage.

**Figure 33:**
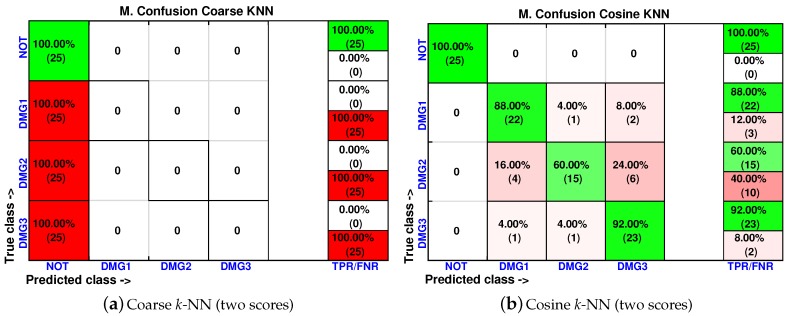
Confusion matrix using coarse *k*-NN (**a**) and cosine *k*-NN (**b**) when the feature vector is formed by the first two principal components.

In Figure 33, we have also summarized the results of the coarse *k*-NN and cosine *k*-NN, which are the classifiers with the lowest accuracy in the classification approach. In particular, the coarse *k*-NN classifies all of the structures with damage as that are undamaged, therefore making this strategy impractical to detect and classify damage.

**Figure 34:**
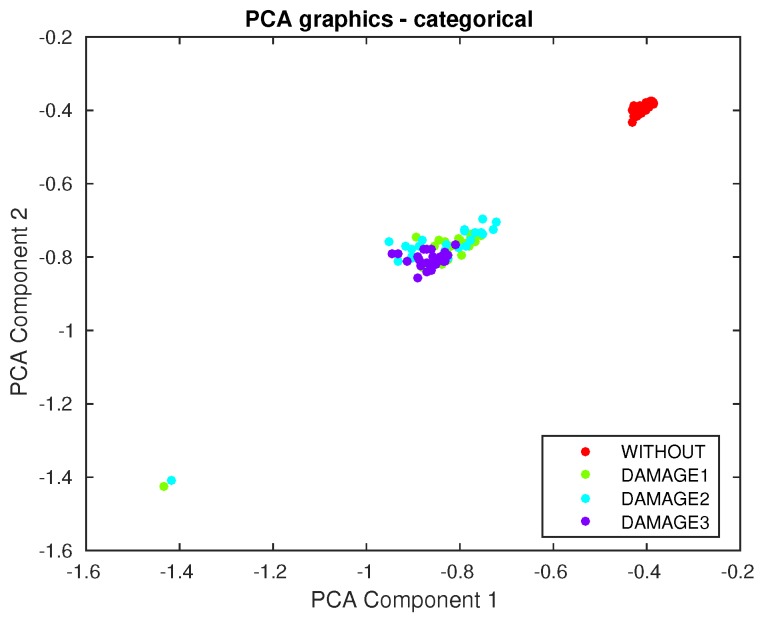
First principal component versus second principal component in the composite plate described in Section 4.3.

Finally, the first principal component versus the second principal component are plotted in Figure 34. It can be observed that, again, a clear separation exists between the composite plate with no damage and the plate with the different kinds of damage. However, it is not possible to separate or classify the different damages, therefore showing the clear benefit of the classifiers used in this work.

## 5. Concluding Remarks

In this contribution, a method to inspect a structure and evaluate possible damage with a piezoelectric sensor network and a machine learning approach is introduced. Results from three specimens—an aluminum rectangular profile, an aluminum plate and a composite plate—showed that just two scores were enough to detect and classify all of the structural states with a very high accuracy. In addition, it is possible to conclude that the best results were obtained with fine *k*-NN and weighted *k*-NN, since the number of correct decisions fluctuate between 85% and 93%. It is worth remarking that for both the fine *k*-NN and weighted *k*-NN and for all three specimens, the structure with no damage is correctly classified in the totality of the cases.

Some features to highlight in the structural health monitoring system are: (i) the methodology uses data-driven approaches and no physical models; this element allows one to determine directly from the data the presence of damage and to determine what kind of damage; (ii) this is a multivariable approach, in the sense that in the analysis, there are measurements from all of the sensors distributed along the structure; and finally, (iii) the approach is based on sensor data fusion. This element is key to obtain a final pattern by merging the results from each actuation phase. This element allows a simplified analysis in larger structures with a large number of sensors.

Another aspect in the methodology that has to be highlighted is the development of a new data organization scheme. This scheme allows the sensor data fusion to perform by offering the opportunity to develop the analysis of the structures in online mode, since one measurement is not related with the others, and the methodology is able to offer results immediately, as soon as they are computed. One of the possible problems with the system is the computational burden of the procedure if the calculations are to be performed in portable equipment.

The piezoelectric active system has allowed inspecting the structures under diagnosis by applying and collecting the signals propagated through the structure, and the sensor data fusion provides robustness to the system given that it allows one to have information from different locations of the structure. This procedure, however, shows some difficulties if the damage is not placed in the exact same location. Further developments will deal with these issues, where it seems possible to avoid these placement problems, training the machines, to create the baseline, with enough experiments.

## Abbreviations

The following abbreviations are used in this manuscript:

*k*-NN	*k*-nearest neighbors algorithm
PCA	Principal component analysis
PZT	Lead-zirconate-titanate
SHM	Structural health monitoring

## References

[1] Anaya M., Tibaduiza D., Torres M.A., Pozo F., Ruiz M., Mujica L.E., Rodellar J., Fritzen C.P. (2014). Data-driven methodology to detect and classify structural changes under temperature variations. Smart Mater. Struct..

[2] Tibaduiza D., Anaya M., Forero E., Castro R., Pozo F. (2016). A sensor fault detection methodology applied to piezoelectric active systems in structural health monitoring applications. IOP Conf. Ser. Mater. Sci. Eng..

[3] Buethe I., Fritzen C.P. Investigation on sensor fault effects of piezoelectric transducers on wave propagation and impedance measurements. Proceedings of the 2013 COMSOL Conference.

[4] Gharibnezhad F. (2014). Robust Damage Detection in Smart Structures. Ph.D. Thesis.

[5] Dervilis N., Worden K., Cross E.J. (2015). On robust regression analysis as a means of exploring environmental and operational conditions for SHM data. J. Sound Vib..

[6] Zhang H., Guo J., Xie X., Bie R., Sun Y. Environmental Effect Removal Based Structural Health Monitoring in the Internet of Things. Proceedings of the 2013 Seventh International Conference on Innovative Mobile and Internet Services in Ubiquitous Computing (IMIS).

[7] Langone R., Reynders E., Mehrkanoon S., Suykens J.A.K. (2017). Automated structural health monitoring based on adaptive kernel spectral clustering. Mech. Syst. Signal Process..

[8] He Q.P., Wang J. (2007). Fault detection using the *k*-nearest neighbor rule for semiconductor manufacturing processes. IEEE Trans. Semicond. Manuf..

[9] Mulligan K.R., Quaegebeur N., Ostiguy P.-C., Masson P., Letourneau S. (2012). Comparison of metrics to monitor and compensate for piezoceramic debonding in structural health monitoring. Struct. Health Monit..

[10] Gui G., Pan H., Lin Z., Li Y., Yuan Z. (2017). Data-driven support vector machine with optimization techniques for structural health monitoring and damage detection. KSCE J. Civ. Eng..

[11] Nick W., Shelton J., Asamene K., Esterline A. A Study of Supervised Machine Learning Techniques for Structural Health Monitoring. Proceedings of the 26th Modern Artificial Intelligence and Cognitive Science Conference (MAICS 2015).

[12] Torres M.A., Buethe I., Tibaduiza D., Rodellar J., Fritzen C.P. (2013). Damage detection and classification in pipework using acousto-ultrasonics and non-linear data-driven modeling. J. Civ. Struct. Health Monit..

[13] Tibaduiza D.A., Mujica L.E., Anaya M., Rodellar J., Güemes A. Independent component analysis for detecting damages on aircraft wing skeleton. Proceedings of the 5th European Conference on Structural Control (EACS 2012).

[14] Gautschi G. (2002). Piezoelectric Sensorics: Force Strain Pressure Acceleration and Acoustic Emission Sensors Materials and Amplifiers.

[15] Anaya M. (2016). Design and Validation of a Structural Health Monitoring System Based on Bio-inspired Algorithms. Ph.D. Thesis.

[16] Jollife I. (2002). Principal Component Analysis.

[17] Anaya M., Tibaduiza D.A., Pozo F. (2015). A bioinspired methodology based on an artificial immune system for damage detection in structural health monitoring. Shock Vib..

[18] Tibaduiza D.A. (2012). Design and Validation of a Structural Health Monitoring System for Aeronautical Structures. Ph.D. Thesis.

[19] Jeong D.H., Ziemkiewicz C., Fisher B., Ribarsky W., Chang R. (2009). iPCA: An interactive system for PCA based visual analytics. Comput. Graph. Forum.

[20] Pozo F., Vidal Y. (2016). Wind turbine fault detection through principal component analysis and statistical hypothesis testing. Energies.

[21] Farrar C., Worden K. (2012). Structural Health Monitoring: A Machine Learning Perspective.

[22] Ciang C.C., Lee J.-R., Bang H.-J. (2008). Structural health monitoring for a wind turbine system: A review of damage detection methods. Meas. Sci. Technol..

[23] Cover T.M., Hart P.E. (1967). Nearest neighbor pattern classification. IEEE Trans. Inf. Theory.

[24] Dhanabal S., Chandramathi S. (2011). A review of various K-nearest neighbor query processing techniques. Int. J. Comput. Appl. Technol..

[25] Yang J., Sun Z., Chen Y. (2016). Fault detection using the clustering-*k*NN rule for gas sensor arrays. Sensors.

[26] Johnson J., Yadav A. Fault Detection and Classification Technique for HVDC Transmission Lines Using *k*NN. Proceedings of the International Conference on ICT for Sustainable Development (ICT4SD).

[27] (2015). Statistics and Machine Learning Toolbox for Matlab.

[28] Deng Z., Zhu X., Cheng D., Zong M., Zhang S. (2016). Efficient *k*NN classification algorithm for big data. Neurocomputing.

[29] Oh S., Byon Y., Yeo H. (2016). Improvement of search strategy with *k*-nearest neighbors approach for traffic state prediction. IEEE Trans. Intell. Transp. Syst..

[30] Tibaduiza D.A., Mujica L.E., Rodellar J. (2012). Damage classification in structural health monitoring using principal component analysis and self-organizing maps. Struct. Control Health Monit..

[31] Mujica L.E., Ruiz M., Pozo F., Rodellar J., Güemes A. (2014). A structural damage detection indicator based on principal component analysis and statistical hypothesis testing. Smart Mater. Struct..

[32] Pozo F., Arruga I., Mujica L.E., Ruiz M., Podivilova E. (2016). Detection of structural changes through principal component analysis and multivariate statistical inference. Struct. Health Monit..

